# Supplementary material for: Optimization of Cas9 activity through the addition of cytosine extensions to single-guide RNAs
Source: Nat Biomed Eng. 2023 Apr 10;7(5):672–91. doi: 10.1038/s41551-023-01011-7 (PMC10195680; doi:10.1038/s41551-023-01011-7)
Supplement: Supplementary file 1 — Reporting Summary [file 41551_2023_1011_MOESM1_ESM.pdf]

nature research

Corresponding author(s): Masaki Kawamata, Hiroshi I. Suzuki, Atsushi Suzuki

Last updated by author(s): Jan 16, 2023

Reporting Summary

Nature Research wishes to improve the reproducibility of the work that we publish. This form provides structure for consistency and transparency in reporting. For further information on Nature Research policies, see our Editorial Policies and the Editorial Policy Checklist.

Statistics

For all statistical analyses, confirm that the following items are present in the figure legend, table legend, main text, or Methods section.

n/a	Confirmed	
☐	☒	The exact sample size (n) for each experimental group/condition, given as a discrete number and unit of measurement
☐	☒	A statement on whether measurements were taken from distinct samples or whether the same sample was measured repeatedly
☐	☒	The statistical test(s) used AND whether they are one- or two-sided<br>*Only common tests should be described solely by name; describe more complex techniques in the Methods section.*
☐	☒	A description of all covariates tested
☐	☒	A description of any assumptions or corrections, such as tests of normality and adjustment for multiple comparisons
☐	☒	A full description of the statistical parameters including central tendency (e.g. means) or other basic estimates (e.g. regression coefficient) AND variation (e.g. standard deviation) or associated estimates of uncertainty (e.g. confidence intervals)
☐	☒	For null hypothesis testing, the test statistic (e.g. F, t, r) with confidence intervals, effect sizes, degrees of freedom and P value noted<br>*Give P values as exact values whenever suitable.*
☒	☐	For Bayesian analysis, information on the choice of priors and Markov chain Monte Carlo settings
☒	☐	For hierarchical and complex designs, identification of the appropriate level for tests and full reporting of outcomes
☐	☒	Estimates of effect sizes (e.g. Cohen's d, Pearson's r), indicating how they were calculated

Our web collection on statistics for biologists contains articles on many of the points above.

Software and code

Policy information about availability of computer code

Data collection	Fluorescent microscopes (BZ-X800, Keyence and IX73, Olympus); Luminescent image analyzer (LAS-3000, FUJIFILM); Fluorescence stereo microscope (M165FC, Leica); Real-Time System (CFX Connect, BIO-RAD).
Data analysis	R version 3.2.1, JMP 14.2.0., FIJI, Graphpad Prism 8.4.3, Microsoft Excel v16. Measurement application software (Keyence, BZ-H4M).

For manuscripts utilizing custom algorithms or software that are central to the research but not yet described in published literature, software must be made available to editors and reviewers. We strongly encourage code deposition in a community repository (e.g. GitHub). See the Nature Research guidelines for submitting code & software for further information.

Data

Policy information about availability of data

All manuscripts must include a data availability statement. This statement should provide the following information, where applicable:

- Accession codes, unique identifiers, or web links for publicly available datasets
- A list of figures that have associated raw data
- A description of any restrictions on data availability

The main data supporting the results in this study are available within the paper and its Supplementary Information. Source data for the figures are provided with this paper. All data generated in this study, are available from the authors on reasonable request.

Field-specific reporting

Please select the one below that is the best fit for your research. If you are not sure, read the appropriate sections before making your selection.

☒ Life sciences ☐ Behavioural & social sciences ☐ Ecological, evolutionary & environmental sciences

For a reference copy of the document with all sections, see nature.com/documents/nr-reporting-summary-flat.pdf

Life sciences study design

All studies must disclose on these points even when the disclosure is negative.

Sample size	We did not use a statistical method to determine sample sizes. We chose sample size (at least n = 3) on the basis of prior experience.
Data exclusions	No data were excluded.
Replication	All attempts to reproduce the results were successful.
Randomization	Randomization was not performed. Sample randomization is not applicable for cell-culture experiments where batches of homogeneous cultures can be tested in parallel.
Blinding	The investigators were not blinded to sample identity. We did not perform any analysis entailing subjective group allocation.

Reporting for specific materials, systems and methods

We require information from authors about some types of materials, experimental systems and methods used in many studies. Here, indicate whether each material, system or method listed is relevant to your study. If you are not sure if a list item applies to your research, read the appropriate section before selecting a response.

Materials & experimental systems

n/a	Involved in the study	
☐	☒	Antibodies
☐	☒	Eukaryotic cell lines
☒	☐	Palaeontology and archaeology
☐	☒	Animals and other organisms
☒	☐	Human research participants
☒	☐	Clinical data
☒	☐	Dual use research of concern

Methods

n/a	Involved in the study	
☒	☐	ChIP-seq
☒	☐	Flow cytometry
☒	☐	MRI-based neuroimaging

Antibodies

Antibodies used	Rabbit polyclonal p53 antibody (Santa Cruz, Catalog number SC-6243, Clone name FL-393, Lot number I0705); Rabbit monoclonal pSmad1/5/8 antibody (Cell Signaling Technology, Catalog number 13820, Clone name D5B10, Lot number 1); Alexa Fluor 488 donkey anti-rabbit secondary antibody IgG (H+L) (Thermo Scientific, Catalog number A21206, Lot number 1796375).
Validation	The validation of the p53 and pSmad1/5/8 antibodies in human cells is stated in the manufacture's website and they have been repeatedly used in previous reports.

Eukaryotic cell lines

Policy information about cell lines

Cell line source(s)	Mouse ESC lines (B6-5-2, B6-D2-4, and R26R YFP/+ mESC) were established for this study. Human iPS cell lines (409B2, HPS0076, and FOP, HPS0376) were provided by the RIKEN BRC. HEK293T cells were kindly provided by Dr. Miyoshi (Keio University). Human adipose-derived stem cells (hADSCs) were purchased (Thermo Fisher Scientific).
Authentication	Cell-line authentication was performed based on cell morphology, growth condition, and specific properties.
Mycoplasma contamination	All cell lines tested were negative for mycoplasma contamination.
Commonly misidentified lines (See ICLAC register)	No commonly misidentified cell lines were used.

Animals and other organisms

Policy information about studies involving animals; ARRIVE guidelines recommended for reporting animal research

Laboratory animals	C57BL/6 mice, male and female (Clea Japan, Tokyo, Japan), ICR mice, male and female (Clea Japan, Tokyo, Japan),R26R YFP/YFP mice, male (a gift from Frank Costantini, Columbia University, New York, NY) and Cdh1-AIMS mice (generated in this work) were used.
Wild animals	The study did not involve wild animals.
Field-collected samples	The study did not involve samples collected from the field.
Ethics oversight	The study were approved by the Kyushu University Animal Experiment Committee, and the care and use of the animals were performed in accordance with institutional guidelines.

Note that full information on the approval of the study protocol must also be provided in the manuscript.
