# Supplementary material for: Optimization of Cas9 activity through the addition of cytosine extensions to single-guide RNAs
Source: Nat Biomed Eng. 2023 Apr 10;7(5):672–91. doi: 10.1038/s41551-023-01011-7 (PMC10195680; doi:10.1038/s41551-023-01011-7)
Supplement: Supplementary file 2 [file 41551_2023_1011_MOESM5_ESM.docx]

Supplementary Table 3. Information of vector construction, genotyping, and oligonucleotides.

Targeting vector construction

Gene	Extension	Direction	Primer
Cdh1 5' arm	*SacII* site	F	CCGCGGCTGATTCCCCCAACTTCAGA
	KpnI site	R	GGTACCGTCGTCCTCACCACCGCCGT
Cdh1 3' arm	*SalI* site	F	GTCGACCGTGTGGCACCATGGGAGATG
		R	GCACCCACACCAAGATACCTG
Tbx3 5' arm		F	GTTCCCCACTCTCTAACTCC
	KpnI site	R	GGTACCATAAGGGGACCCGCTGCAAGA
Tbx3 3' arm	*SalI* site	F	GTCGACCTGCCAGTGCACTTTGTTAG
	NsiI site	R	ATGCATCCTCTACAAGCGCTCAGATA
tdTomato	*KpnI* site + P2A1	F	GGTACCGGAAGCGGAGCTACTAACTTCAGCCTGCTGAAGCAGGCTGGAGACGTGGAGGAGAACCCTGGACCTATGGTGAGCAAGGGCGAGGAGGTCAT
tdTomato and Venus	*KpnI* site + P2A2	F	GGTACCGGGAGTGGGGCCACCAATTTTAGTCTACTAAAACAAGCCGGGGATGTAGAAGAAAATCCCGGGCCCATGGTGAGCAAGGGCGAGGAG
tdTomato	*SalI* site	R	GTCGACGTTGGGCCATGGCGGCCAAGCTT
Venus	*KpnI* site + P2A1	F	GGTACCGGAAGCGGAGCTACTAACTTCAGCCTGCTGAAGCAGGCTGGAGACGTGGAGGAGAACCCTGGACCTATGGTGAGCAAGGGCGAGGAGGTCAT
Venus	*SalI* site	R	GTCGACTTACTTGTACAGCTCGTCCAT
Neo-PolyA	*KpnI* site + P2A1	F	GGTACCGGAAGCGGAGCTACTAACTTCAGCCTGCTGAAGCAGGCTGGAGACGTGGAGGAGAACCCTGGACCTATGGGATCGGCCATTGAACAAGATGG
	SalI site	R	GTCGACCAGCTGGTTCTTTCCGCCTCAG

Genotyping, genomic PCR, and q-PCR

Gene	Application	Direction	Primer
Cdh1 5' arm external	Genotyping for KI	F	AGGCCACACCTCCTAATCCT
tdTomato 5'		R	ACCTTGAAGCGCATGAACTC
tdTomato 3'		F	CACCATCGTGGAACAGTACG
Cdh1 3' arm external		R	GCATGCATAGTGGTCCCTTT
Venus 5'		R	AGATCAGCTTCAGGGTCAGC
Venus 3'		F	ACATGGTCCTGCTGGAGTTC
Cdh1	Genotyping and indel detectior	F	CGACTCTCTGTTGGTGTTCG
		R	TCTGGGGCAGATCATAGCTC
Tbx3 5' arm external	Genotyping for KI	F	TAGTAGGCGGTGGAGGAGAA
Tbx3 3' arm external		R	CTAATGGTCCAACAGGCACA
Tbx3	Genotyping and indel detectior	F	ACTCGGGGTCGGAACTGAAC
		R	CCTGGCTACCTTGTTCTTC
Tbx3	Genotyping and indel detectior	F	ACTCGGGGTCGGAACTGAAC
		R	CCTGGCTACCTTGTTCTTC
Rosa26		F	CGTGTTCGTGCAAGTTGAGT
		R	CTCCACTGCAGCTCCCTTAC
Alb		F	GGAGAGCCACTTGCCTCATA
		R	TTGTGGTCTTTCCCTCATCC
Acvr1		F	AGTTGCTGGAAATCCTGTGG
		R	TTCACGCTAGGCATTGTCTG
ACVR1		F1	GCTGCCCTTCATGTGAGTTA
		R1	CATCATCCCAAAGGGTCCAC
		F2	CAGATTGCGCCATTGAACTG
		R2	CATCATCCCAAAGGGTCCAC
EMX1		F	AAAACCACCCTTCTCTCTGGC
		R	GGAGATTGGAGACACGGAGAG
HCN1		F	TCTCTCCTTCAACTCATGACCAGCT
		R	ATCTGCACATGTATGTACAGGAGTCAT
MFAP1		F	TGAAATCTCACCTGGGCGAGA
		R	TGCAGTCTGCCTTTTTGGGG
VEGFA1		F	ACACAGCTTCCCGTTCTCAG
		R	AGGGAGCAGGAAAGTGAGGT
VEGFA1for sequencing in hADSC (PicoPLEX)		F	AAGCCCATTCCCTCTTTAGC
		R	CTGAGAGCCGTTCCCTCTTT
MAX		F	GAGGGGGAAGTCACCGACAA
		R	TACCCGGGCCGTCTGTTAGA
COMDA		F	TCTGTCACCACACAGTTACCACC
		R	GTTGCCTGGGGATGGGGTAT
Venus allele (Pr.1, Fig. 5g)	Compound heterozygous muta	F	ACATGGTCCTGCTGGAGTTC
tdTomato allele (Pr.2, Fig. 5g)		F	CCTGTTCCTGTACGGCATGG
Tbx3 genomic locus (Pr.3, Fig. 5g)		R	TCTGGGGCAGATCATAGCTC
ASCL1 (CRISPRa)	q-PCR	F	CGCGGCCAACAAGAAGATG
		R	CGACGAGTAGGATGAGACCG
TTN (CRISPRa)		F	TGTTGCCACTGGTGCTAAAG
		R	ACAGCAGTCTTCTCCGCTTC
BRCA1 (CRISPRi)		F	CTCAAGGAACCAGGGATGAA
		R	GCTGTAATGAGCTGGCATGA
CXCR4 (CRISPRi)		F	ACTACACCGAGGAAATGGGCT
		R	CCCACAATGCCAGTTAAGAAGA
GAPDH		F	GCACCGTCAAGGCTGAGAAC
		R	TGGTGAAGACGCCAGTGGA

gRNA and ssODNs

For [C]sgRNA PX459 plasmids

sgRNA name	Extension	Direction	oligo
P2A1-sgRNA1	[15C]	F	CACCGCCCCCCCCCCCCCCCTAACTTCAGCCTGCTGAAGC
		R	AAACGCTTCAGCAGGCTGAAGTTAGGGGGGGGGGGGGGGC
	[15G]	F	CACCGGGGGGGGGGGGGGGGTAACTTCAGCCTGCTGAAGC
		R	AAACGCTTCAGCAGGCTGAAGTTACCCCCCCCCCCCCCCC
	[15A]	F	CACCGAAAAAAAAAAAAAAATAACTTCAGCCTGCTGAAGC
		R	AAACGCTTCAGCAGGCTGAAGTTATTTTTTTTTTTTTTTC
	[15T]	F	CACCGTTTTTTTTTTTTTTTTAACTTCAGCCTGCTGAAGC
		R	AAACGCTTCAGCAGGCTGAAGTTAAAAAAAAAAAAAAAAC
P2A1-sgRNA1		F	CACCGTAACTTCAGCCTGCTGAAGC
		R	AAACGCTTCAGCAGGCTGAAGTTAC
P2A1-sgRNA2		F	CACCGCGTCTCCAGCCTGCTTCAGC
		R	AAACGCTGAAGCAGGCTGGAGACGC
P2A1-sgRNA3		F	CACCGCTGAAGCAGGCTGGAGACG
		R	AAACCGTCTCCAGCCTGCTTCAGC
P2A2-sgRNA4		F	CACCGTAGTCTACTAAAACAAGCCG
		R	AAACCGGCTTGTTTTAGTAGACTAC
P2A2-sgRNA5		F	CACCGGGATGTAGAAGAAAATCCC
		R	AAACGGGATTTTCTTCTACATCCC
P2A2-sgRNA6		F	CACCGTTTTAGTAGACTAAAATTGG
		R	AAACCCAATTTTAGTCTACTAAAAC
P2A1-sgRNA1	[0C]	F	CACCGTAACTTCAGCCTGCTGAAGC
		R	AAACGCTTCAGCAGGCTGAAGTTAC
	[5C]	F	CACCGCCCCCTAACTTCAGCCTGCTGAAGC
		R	AAACGCTTCAGCAGGCTGAAGTTAGGGGGC
	[10C]	F	CACCGCCCCCCCCCCTAACTTCAGCCTGCTGAAGC
		R	AAACGCTTCAGCAGGCTGAAGTTAGGGGGGGGGGC
	[15C]	F	CACCGCCCCCCCCCCCCCCCTAACTTCAGCCTGCTGAAGC
		R	AAACGCTTCAGCAGGCTGAAGTTAGGGGGGGGGGGGGGGC
	[20C]	F	CACCGCCCCCCCCCCCCCCCCCCCCTAACTTCAGCCTGCTGAAGC
		R	AAACGCTTCAGCAGGCTGAAGTTAGGGGGGGGGGGGGGGGGGGGC
	[25C]	F	CACCGCCCCCCCCCCCCCCCCCCCCCCCCCTAACTTCAGCCTGCTGAAGC
		R	AAACGCTTCAGCAGGCTGAAGTTAGGGGGGGGGGGGGGGGGGGGGGGGGC
	[30C]	F	CACCGCCCCCCCCCCCCCCCCCCCCCCCCCCCCCCTAACTTCAGCCTGCTGAAGC
		R	AAACGCTTCAGCAGGCTGAAGTTAGGGGGGGGGGGGGGGGGGGGGGGGGGGGGGC
	[35C]	F	CACCGCCCCCCCCCCCCCCCCCCCCCCCCCCCCCCCCCCCTAACTTCAGCCTGCTGAAGC
		R	AAACGCTTCAGCAGGCTGAAGTTAGGGGGGGGGGGGGGGGGGGGGGGGGGGGGGGGGGGC
	[40C]	F	CACCGCCCCCCCCCCCCCCCCCCCCCCCCCCCCCCCCCCCCCCCCTAACTTCAGCCTGCTGAAGC
		R	AAACGCTTCAGCAGGCTGAAGTTAGGGGGGGGGGGGGGGGGGGGGGGGGGGGGGGGGGGGGGGGC
P2A2-sgRNA4	[0C]	F	CACCGTAGTCTACTAAAACAAGCCG
		R	AAACCGGCTTGTTTTAGTAGACTAC
	[5C]	F	CACCGCCCCCTAGTCTACTAAAACAAGCCG
		R	AAACCGGCTTGTTTTAGTAGACTAGGGGGC
	[10C]	F	CACCGCCCCCCCCCCTAGTCTACTAAAACAAGCCG
		R	AAACCGGCTTGTTTTAGTAGACTAGGGGGGGGGGC
	[15C]	F	CACCGCCCCCCCCCCCCCCCTAGTCTACTAAAACAAGCCG
		R	AAACCGGCTTGTTTTAGTAGACTAGGGGGGGGGGGGGGGC
	[20C]	F	CACCGCCCCCCCCCCCCCCCCCCCCTAGTCTACTAAAACAAGCCG
		R	AAACCGGCTTGTTTTAGTAGACTAGGGGGGGGGGGGGGGGGGGGC
	[25C]	F	CACCGCCCCCCCCCCCCCCCCCCCCCCCCCTAGTCTACTAAAACAAGCCG
		R	AAACCGGCTTGTTTTAGTAGACTAGGGGGGGGGGGGGGGGGGGGGGGGGC
	[30C]	F	CACCGCCCCCCCCCCCCCCCCCCCCCCCCCCCCCCTAGTCTACTAAAACAAGCCG
		R	AAACCGGCTTGTTTTAGTAGACTAGGGGGGGGGGGGGGGGGGGGGGGGGGGGGGC
	[35C]	F	CACCGCCCCCCCCCCCCCCCCCCCCCCCCCCCCCCCCCCCTAGTCTACTAAAACAAGCCG
		R	AAACCGGCTTGTTTTAGTAGACTAGGGGGGGGGGGGGGGGGGGGGGGGGGGGGGGGGGGC
	[40C]	F	CACCGCCCCCCCCCCCCCCCCCCCCCCCCCCCCCCCCCCCCCCCCTAGTCTACTAAAACAAGCCG
		R	AAACCGGCTTGTTTTAGTAGACTAGGGGGGGGGGGGGGGGGGGGGGGGGGGGGGGGGGGGGGGGC
Ligation into *BpiI* site of PX459	[5C (3A)] linker	F	CACCGCCACCGGGTCTTCGAGAAGACCT
(Extended Data Fig. 2b)		R	AAACAGGTCTTCTCGAAGACCCGGTGGC
	[10C (8A)] linker	F	CACCGCCCCCCCACCGGGTCTTCGAGAAGACCT
		R	AAACAGGTCTTCTCGAAGACCCGGTGGGGGGGC
	[15C (13A)] linker	F	CACCGCCCCCCCCCCCCACCGGGTCTTCGAGAAGACCT
		R	AAACAGGTCTTCTCGAAGACCCGGTGGGGGGGGGGGGC
	[20C (18A)] linker	F	CACCGCCCCCCCCCCCCCCCCCACCGGGTCTTCGAGAAGACCT
		R	AAACAGGTCTTCTCGAAGACCCGGTGGGGGGGGGGGGGGGGGC
	[25C (23A)] linker	F	CACCGCCCCCCCCCCCCCCCCCCCCCCACCGGGTCTTCGAGAAGACCT
		R	AAACAGGTCTTCTCGAAGACCCGGTGGGGGGGGGGGGGGGGGGGGGGC
	[30C (28A)] linker	F	CACCGCCCCCCCCCCCCCCCCCCCCCCCCCCCACCGGGTCTTCGAGAAGACCT
		R	AAACAGGTCTTCTCGAAGACCCGGTGGGGGGGGGGGGGGGGGGGGGGGGGGGC
Cdh1-sgRNA1		F	CACCGTCTCCCCCGTGTGGCACCAT
		R	AAACATGGTGCCACACGGGGGAGAC
Cdh1-sgRNA2		F	CACCGAGTCTCTCTTCTACACACTC
		R	AAACGAGTGTGTAGAAGAGAGACTC
Tbx3-sgRNA1		F	CACCGACTGGCAGATTTGACCAAAC
		R	AAACGTTTGGTCAAATCTGCCAGTC
Alb-sgRNA1		F	CACCGACTCCATCACTTAAGGCCCC
		R	GGGGCCTTAAGTGATGGAGT
Rosa26-sgRNA1		F	AGACCTCCATCGCGCACTCC
		R	GGAGTGCGCGATGGAGGTCT
Acvr1-sgRNA1 (WT)		F	CACCGGCTCGCCAGATAACCCTGT
		R	AAACACAGGGTTATCTGGCGAGCC
ACVR1-sgRNA1 (R206H)		F	CACCGGCTCACCAGATTACACTGT
		R	AAACACAGTGTAATCTGGTGAGCC
ACVR1-sgRNA1 (WT)		F	CACCGGCTCGCCAGATTACACTGT
		R	AAACACAGTGTAATCTGGCGAGCC
EMX1 on-target-sgRNA1		F	CACCGAGTCCGAGCAGAAGAAGAA
		R	AAACTTCTTCTTCTGCTCGGACTC
EMX1 off-target1-sgRNA1 (HCN1)		F	TCTCTCCTTCAACTCATGACCAGCT
		R	ATCTGCACATGTATGTACAGGAGTCAT
EMX1 off-target1-sgRNA1 (MFAP1)		F	TGAAATCTCACCTGGGCGAGA
		R	TGCAGTCTGCCTTTTTGGGG
VEGFA1 on-target-sgRNA1		F	CACCGGTGAGTGAGTGTGTGCGTG
		R	AAACCACGCACACACTCACTCACC
VEGFA1 off-target1-sgRNA1 (MAX)		F	GAGGGGGAAGTCACCGACAA
		R	TACCCGGGCCGTCTGTTAGA
VEGFA1 off-target2-sgRNA1 (CAMDA)		F	TCTGTCACCACACAGTTACCACC
		R	GTTGCCTGGGGATGGGGTAT
VEGFA1 off-target3-sgRNA1		F	TCCTTTGAGGTTCATCCCCC
		R	CCAATCCAGGATGATTCCGC
VEGFA1 off-target4-sgRNA1		F	CTCCAGAGCCTGGCCTACCA
		R	CCCTCTCCGGAAGTGCCTTG
VEGFA1 off-target5-sgRNA1		F	TGTGGAGGGTGGGACCTGGT
		R	ACAGTGAGGTGCGGTCTTTGGG
VEGFA1 off-target6-sgRNA1		F	GCAGGCAAGCTGTCAAGGGT
		R	CACCGACACACCCACTCACC
VEGFA1 off-target7-sgRNA1		F	GGGCCTCCCTGCTGGTTCTC
		R	GCTGCCGTCCGAACCCAAGA
VEGFA1 off-target8-sgRNA1		F	GACACCCCACACACTCTCATGC
		R	TGAATCCCTTCACCCCCAAG

Acvr1 R206H KI ssODN	Extra nucleotides		GGCCGCCTACTAGATCACTCGTGTACATCAGGAAGTGGCTCCGGTCTTCCTTTCCTGGTAC<br>AGAGAACGGTGGCTCACCAGATAACCCTGTTGGAGTGTGTCGGTAATTCTATGTTTCCCTTC<br>CTTTTGGGCAGCAGGCAATGTTGGCCTGCA
ACVR1 for R206H correction ssODN	Extra nucleotides		GGCCGCATTTATTGGATCATTCGTGTACATCAGGAAGTGGCTCTGGTCTTCCTTTTCTGGTAC<br>AAAGAACAGTGGCGCGCCAGATTACACTGTTGGAGTGTGTCGGTAATTCTTTTTTTTCCTTTC<br>TTTGTGGGTAATATGCAATGTTACCTGCA
ACVR1 for R206H KI ssODN	Extra nucleotides		**GGCCGC**ATTTATTGGATCATTCGTGTACATCAGGAAGTGGCTCTGGTCTTCCTTTTCTGGTAC<br>AAAGAACAGTGGCTCACCAGATTACACTGTTGGAGTGTGTCGGTAATTCTTTTTTTTCCTTTCT<br>TTGTGGGTAATATGCAATGTTA**CCTGCA**
ACVR1 for 3 bp replacement (+*MscI* site) ssODN	Extra nucleotides		GGCCGCATTTATTGGATCATTCGTGTACATCAGGAAGTGGCTCTGGTCTTCCTTTTCTGGTAC<br>AAAGAACAGTGGCCAACCAGATTACACTGTTGGAGTGTGTCGGTAATTCTTTTTTTTCCTTTC<br>TTTGTGGGTAATATGCAATGTTACCTGCA
PCR amplicon for in vitro cleavage		F	ACTCGGGGTCGGAACTGAAC
		R	GTCCTCCTTGAAGTCGATGC
Template amplification for IVT	T7 promoter	F	GCGGCCTCTAATACGACTCACTATAGGGTAACTTCAGCCTGCTGAAGC
	T7 promoter+10C	F	GCGGCCTCTAATACGACTCACTATAGGGCCCCCCCCCCTAACTTCAGCCTGCTGAAGC
	T7 promoter+25C	F	GCGGCCTCTAATACGACTCACTATAGGGCCCCCCCCCCCCCCCCCCCCCCCCCTAACTTCAGCCTGCTGAAGC
		R	AAAAAAGCACCGACTCGGTGCCAC
mU6 probe for Northern blot (93 bp)		F	CTCGCTTCGGCAGCACATATACT
		R	ACGCTTCACGAATTTGCGTGTC
Priming for mU6 cDNA synthesis		R	CTTCGATGTCGACTCGAGTCAAAAAGCACCGACTCGGTGCCAC
Probe for Northern blot		F	GTTTTAGAGCTAGAAATAGCAAGTTAA
		R	AAGCACCGACTCGGTGCCAC
Probe for gel-shift assay		F	GAGGGCCTATTTCCCATGAT
		R	TTGGTACCGCCATTTGTCTGCAGAATTGGC
Synthetic gRNA for gel-shift and cleavage assays	P2A1-[0C]sgRNA1		GTAACTTCAGCCTGCTGAAGCGTTTTAGAGCTAGAAATAGCAAGTTAAAATAAGGCTAGTCC<br>GTTATCAACTTGAAAAAGTGGCACCGAGTCGGTGCTTTT
Custom Alt-R gRNA (IDT)	P2A1-[10C]sgRNA1		GCCCCCCCCCCTAACTTCAGCCTGCTGAAGCGTTTTAGAGCTAGAAATAGCAAGTTAAAATA<br>AGGCTAGTCCGTTATCAACTTGAAAAAGTGGCACCGAGTCGGTGCTTTT
	P2A1-[25C]sgRNA1		GCCCCCCCCCCCCCCCCCCCACCCCCTAACTTCAGCCTGCTGAAGCGTTTTAGAGCTAGA<br>AATAGCAAGTTAAAATAAGGCTAGTCCGTTATCAACTTGAAAAAGTGGCACCGAGTCGGTGC<br>TTTT
P2A1-sgRNA1	30nt	F	CACCGCGGAGCTACTAACTTCAGCCTGCTGAAGC
		R	AAACGCTTCAGCAGGCTGAAGTTAGTAGCTCCGC
P2A1-sgRNA1	40nt	F	CACCGGTACCGGAAGCGGAGCTACTAACTTCAGCCTGCTGAAGC
		R	AAACGCTTCAGCAGGCTGAAGTTAGTAGCTCCGCTTCCGGTACC
P2A1-sgRNA2	30nt	F	CACCGTTCTCCTCCACGTCTCCAGCCTGCTTCAGC
		R	AAACGCTGAAGCAGGCTGGAGACGTGGAGGAGAAC
P2A1-sgRNA2	40nt	F	CACCGAGGTCCAGGGTTCTCCTCCACGTCTCCAGCCTGCTTCAGC
		R	AAACGCTGAAGCAGGCTGGAGACGTGGAGGAGAACCCTGGACCTC
CRISPRa			
ASCL1-gRNA	[0C]	F	CACCGCGGGAGAAAGGAACGGGAGG
		R	AAACCCTCCCGTTCCTTTCTCCCGC
	[1C]	F	CACCGCCGGGAGAAAGGAACGGGAGG
		R	AAACCCTCCCGTTCCTTTCTCCCGGC
	[2C]	F	CACCGCCCGGGAGAAAGGAACGGGAGG
		R	AAACCCTCCCGTTCCTTTCTCCCGGGC
	[3C]	F	CACCGCCCCGGGAGAAAGGAACGGGAGG
		R	AAACCCTCCCGTTCCTTTCTCCCGGGGC
	[4C]	F	CACCGCCCCCGGGAGAAAGGAACGGGAGG
		R	AAACCCTCCCGTTCCTTTCTCCCGGGGGC
	[5C]	F	CACCGCCCCCCGGGAGAAAGGAACGGGAGG
		R	AAACCCTCCCGTTCCTTTCTCCCGGGGGGC
	[6C]	F	CACCGCCCCCCCGGGAGAAAGGAACGGGAGG
		R	AAACCCTCCCGTTCCTTTCTCCCGGGGGGGC
	[10C]	F	CACCGCCCCCCCCCCCGGGAGAAAGGAACGGGAGG
		R	AAACCCTCCCGTTCCTTTCTCCCGGGGGGGGGGGC
TTN-gRNA		F	CACCGCCTTGGTGAAGTCTCCTTTG
		R	AAACCAAAGGAGACTTCACCAAGGC
CRISPRi			
BRCA1-gRNA	[0C]	F	CACCGGCTCGCTGAGACTTCCTGGA
		R	AAACTCCAGGAAGTCTCAGCGAGCC
	[1C]	F	CACCGCGCTCGCTGAGACTTCCTGGA
		R	AAACTCCAGGAAGTCTCAGCGAGCGC
	[2C]	F	CACCGCCGCTCGCTGAGACTTCCTGGA
		R	AAACTCCAGGAAGTCTCAGCGAGCGGC
	[3C]	F	CACCGCCCGCTCGCTGAGACTTCCTGGA
		R	AAACTCCAGGAAGTCTCAGCGAGCGGGC
	[4C]	F	CACCGCCCCGCTCGCTGAGACTTCCTGGA
		R	AAACTCCAGGAAGTCTCAGCGAGCGGGGC
	[5C]	F	CACCGCCCCCGCTCGCTGAGACTTCCTGGA
		R	AAACTCCAGGAAGTCTCAGCGAGCGGGGGC
	[6C]	F	CACCGCCCCCCGCTCGCTGAGACTTCCTGGA
		R	AAACTCCAGGAAGTCTCAGCGAGCGGGGGGC
	[7C]	F	CACCGCCCCCCCGCTCGCTGAGACTTCCTGGA
		R	AAACTCCAGGAAGTCTCAGCGAGCGGGGGGGC
CXCR4-gRNA		F	CACCGCAGGTAGCAAAGTGACGCCGA
		R	AAACTCGGCGTCACTTTGCTACCTGC
VP64-T2A-PuroR	synthetic DNA		GCTAGCCGCGCC**GACGCGCTGGACGATTTCGATCTCGACATGCTGGGTTCTGATGCCCTC**<br>**GATGACTTTGACCTGGATATGTTGGGAAGCGACGCATTGGATGACTTTGATCTGGACATG**<br>**CTCGGCTCCGATGCTCTGGACGATTTCGATCTCGATATGTTA**ATCGGTACCGAGGGCAGAG<br>GAAGTCTTCTAACATGCGGTGACGTGGAGGAGAATCCCGGCCCT**ATGACCGAGTACAAGC**<br>**CCACGGTGCGCCTCGCCACCCGCGACGACGTCCCCAGGGCCGTACGCACCCTCGCCGC**<br>**CGCGTTCGCCGACTACCCCGCCACGCGCCACACCGTCGATCCGGACCGCCACATCGAG**<br>**CGGGTCACCGAGCTGCAAGAACTCTTCCTCACGCGCGTCGGGCTCGACATCGGCAAGGT**<br>**GTGGGTCGCGGACGACGGCGCCGCGGTGGCGGTCTGGACCACGCCGGAGAGCGTCGAA**<br>**GCGGGGGCGGTGTTCGCCGAGATCGGCCCGCGCATGGCCGAGTTGAGCGGTTCCCGGCT**<br>**GGCCGCGCAGCAACAGATGGAAGGCCTCCTGGCGCCGCACCGGCCCAAGGAGCCCGC**<br>**GTGGTTCCTGGCCACCGTCGGCGTGTCGCCCGACCACCAGGGCAAGGGTCTGGGCAGC**<br>**GCCGTCGTGCTCCCCGGAGTGGAGGCGGCCGAGCGCGCCGGGGTGCCCGCCTTCCTGG**<br>**AGACCTCCGCGCCCCGCAACCTCCCCTTCTACGAGCGGCTCGGCTTCACCGTCACCGC**<br>**CGACGTCGAGGTGCCCGAAGGACCGCGCACCTGGTGCATGACCCGCAAGCCCGGTGCC**<br>TGACCAGCACACTGGCGGCCGTTACTAGCTTCTGCAGCACGACCGGT
U6-crRNA cloning cassettes (AsCpf1)	synthetic DNA		**ACATGTGAGGGCCTATTTCCCATGATTCCTTCATATTTGCATATACGATACAAGGCTGTTA**<br>**GAGAGATAATTGGAATTAATTTGACTGTAAACACAAAGATATTAGTACAAAATACGTGAC**<br>**GTAGAAAGTAATAATTTCTTGGGTAGTTTGCAGTTTTAAAATTATGTTTTAAAATGGACTAT**<br>**CATATGCTTACCGTAACTTGAAAGTATTTCGATTTCTTGGCTTTATATATCTTGTGGAAAGG**<br>**AC**GAAACACCGGGTCTTCGAGAAGACCTTTTTTTGTTTTAGAGCTAGAAATAGCAAGTTAAAA<br>TAAGGCTAGTCCGTTTTTAGCGCGTGCGCCAATTCTGCAGACAAATGGCTCTAGA
AsCpf1 (P2A2-gRNA7)			
5' end	[0C]	F	CACC**GTAATTTCTACTCTTGTAGATGTCTACTAAAACAAGCCGGGGTTTTATTTT**
		R	AAACAAAATAAAACCCCGGCTTGTTTTAGTAGACATCTACAAGAGTAGAAATTAC
	[5C]	F	CACC**GCCCCCTAATTTCTACTCTTGTAGATGTCTACTAAAACAAGCCGGGGTTTTATTTT**
		R	AAACAAAATAAAACCCCGGCTTGTTTTAGTAGACATCTACAAGAGTAGAAATTAGGGGGC
	[10C]	F	CACC**GCCCCCCCCCCTAATTTCTACTCTTGTAGATGTCTACTAAAACAAGCCGGGGTTTTATTTT**
		R	AAACAAAATAAAACCCCGGCTTGTTTTAGTAGACATCTACAAGAGTAGAAATTAGGGGGGGGGGC
	[15C]	F	CACC**GCCCCCCCCCCCCCCCTAATTTCTACTCTTGTAGATGTCTACTAAAACAAGCCGGGGTTTTATTTT**
		R	AAACAAAATAAAACCCCGGCTTGTTTTAGTAGACATCTACAAGAGTAGAAATTAGGGGGGGGGGGGGGGC
	[20C]	F	CACC**GCCCCCCCCCCCCCCCCCCCCTAATTTCTACTCTTGTAGATGTCTACTAAAACAAGCCGGGGTTTTATTTT**
		R	AAACAAAATAAAACCCCGGCTTGTTTTAGTAGACATCTACAAGAGTAGAAATTAGGGGGGGGGGGGGGGGGGGGC
	[25C]	F	CACC**GTAATTTCTACTCTTGTAGATGTCTACTAAAACAAGCCGGGGCCCCCCCCCCCCCCCCCCCCCCCCCTTTTATTTT**
		R	AAACAAAATAAAAGGGGGGGGGGGGGGGGGGGGGGGGGCCCCGGCTTGTTTTAGTAGACATCTACAAGAGTAGAAATTAC
	[30C]	F	CACC**GCCCCCCCCCCCCCCCCCCCCCCCCCCCCCCTAATTTCTACTCTTGTAGATGTCTACTAAAACAAGCCGGGGTTTTATTTT**
		R	AAACAAAATAAAACCCCGGCTTGTTTTAGTAGACATCTACAAGAGTAGAAATTAGGGGGGGGGGGGGGGGGGGGGGGGGGGGGGC
middle	[10C]	F	CACC**GTAATTTCTACTCTTGTAGATCCCCCCCCCCGTCTACTAAAACAAGCCGGGGTTTTATTTT**
		R	AAACAAAATAAAACCCCGGCTTGTTTTAGTAGACGGGGGGGGGGATCTACAAGAGTAGAAATTAC
	[25C]	F	CACC**GTAATTTCTACTCTTGTAGATCCCCCCCCCCCCCCCCCCCCCCCCCGTCTACTAAAACAAGCCGGGGTTTTATTTT**
		R	AAACAAAATAAAACCCCGGCTTGTTTTAGTAGACGGGGGGGGGGGGGGGGGGGGGGGGGATCTACAAGAGTAGAAATTAC
3' end	[10C]	F	CACC**GTAATTTCTACTCTTGTAGATGTCTACTAAAACAAGCCGGGGCCCCCCCCCCTTTTATTTT**
